# GITR Ligation Improves Anti-PD1-Mediated Restoration of Human MMR-Proficient Colorectal Carcinoma Tumor-Derived T Cells

**DOI:** 10.1016/j.jcmgh.2022.09.007

**Published:** 2022-09-23

**Authors:** Yannick S. Rakké, Lucia Campos Carrascosa, Adriaan A. van Beek, Valeska de Ruiter, Rachelle S. van Gemerden, Michail Doukas, Pascal G. Doornebosch, Maarten Vermaas, Susan ter Borg, Erwin van der Harst, Peter Paul L.O. Coene, Mike Kliffen, Dirk J. Grünhagen, Cornelis Verhoef, Jan N.M. IJzermans, Jaap Kwekkeboom, Dave Sprengers

**Affiliations:** 1 Department of Surgery, Erasmus MC-University Medical Center, Rotterdam, the Netherlands; 2 Department of Gastroenterology and Hepatology, Erasmus MC-University Medical Center, Rotterdam, the Netherlands; 3 Department of Pathology, Erasmus MC-University Medical Center, Rotterdam, the Netherlands; 4 Department of Surgery, IJsselland Hospital, Capelle aan den IJssel, the Netherlands; 5 Pathan BV, Rotterdam, the Netherlands; 6 Department of Surgery, Maasstad Hospital, Rotterdam, the Netherlands; 7 Department of Pathology, Maasstad Hospital, Rotterdam, the Netherlands

**Keywords:** Colorectal carcinoma, Immune Checkpoint Stimulation, Liver metastasis, Microsatellite Stable, TNF Receptor Superfamily, Tumor-Infiltrating Lymphocytes, (a)Th, (activated) helper T cell, (a/r)Treg, (activated/resting) regulatory T cell, CRC, primary colorectal carcinoma, CRLM, colorectal carcinoma-derived liver metastasis, CTL, cytotoxic T lymphocyte, dMMR, mismatch repair deficient, FoxP3, Forkhead box P3, GITR, glucocorticoid-induced tumor necrosis factor receptor-related protein, GITRL, GITR ligand, GzmA/B, granzyme A/B, HCC, hepatocellular carcinoma, ICB, immune checkpoint blockade, IFN-γ, interferon gamma, LAG3, lymphocyte-activation gene 3, MFI, median fluorescence intensity, MSI(-H/-L), microsatellite instable (-high/-low), MSS, microsatellite stable, PBMC, peripheral blood mononuclear cells, PD1^(hi)^, programmed cell death protein 1(-high), pMMR, mismatch repair proficient, TCR, T-cell receptor, Tex, exhausted T cell, Th1, T helper 1, TIL, tumor-infiltrating lymphocytes, TME, tumor microenvironment, TNFRSF, tumor necrosis factor receptor superfamily, TNF-α, tumor necrosis factor alpha

## Abstract

**Background & Aims:**

In contrast to mismatch repair deficient colorectal carcinoma (CRC), MMR proficient (pMMR) CRC does not respond to immune checkpoint blockade. We studied immune checkpoint stimulation via glucocorticoid-induced tumor necrosis factor receptor-related protein (GITR) on ex vivo functionality of human tumor-infiltrating lymphocytes (TIL) isolated from pMMR primary CRC and liver metastases (CRLM).

**Methods:**

Using lymphocytes from resected tumor, adjacent tissues, and peripheral blood mononuclear cells (PBMC) of 132 pMMR primary CRC or CRLM patients, we determined GITR expression and the in vitro T-cell agonistic activity of recombinant GITR ligation.

**Results:**

Here, we show that GITR was overexpressed on TIL when compared with other stimulatory immune checkpoints (4-1BB, OX40). Its expression was enhanced in TIL compared with PBMC and adjacent tissues. Among CD4^+^ TIL, GITR expression was primarily expressed by CD45RA^-^ FoxP3^hi^ activated regulatory T cells. Within CD8^+^ TIL, GITR was predominantly expressed on functionally exhausted and putative tumor-reactive CD103^+^ CD39^+^ TIL. Strikingly, recombinant GITRL reinvigorated ex vivo TIL responses by significantly enhancing CD4^+^ and CD8^+^ TIL numbers. Dual treatment with GITRL and nivolumab (anti-PD1) enhanced CD8^+^ TIL expansion compared with GITRL monotherapy. Moreover, GITRL/anti-PD1 dual therapy further improved anti-PD1-mediated reinvigoration of interferon gamma secretion by exhausted CD8 TIL from primary CRC.

**Conclusions:**

GITR is overexpressed on CD4^+^ and CD8^+^ TIL from pMMR CRC and CRLM. Agonistic targeting of GITR enhances ex vivo human TIL functionality and may therefore be a promising approach for novel monotherapy or combined immunotherapies in primary pMRR CRC and CRLM.

**Summary**

We demonstrate that GITR is predominantly expressed on human pMMR CRC- and CRLM-derived TIL. We show that GITR-mediated checkpoint stimulation ameliorates TIL functionality and anti-PD1-mediated TIL reinvigoration, thereby providing rationale for immunotherapies targeting GITR in pMMR CRC and CRLM patients.

Colorectal cancer (CRC) is the second and third most common cancer worldwide in women and men, respectively.^1^ In 2020, 1.9 million newly diagnosed patients and 935,000 disease-related deaths were reported, letting CRC account for 10% of total cancer incidence and mortality annually.^1^ Even though recent diagnostic and treatment regimens have enhanced overall survival rates significantly, there remains a need for further improvement.^2^ Extensive analyses on genomic, epigenomic, and transcriptomic CRC features have extended our understanding on CRC biology and its stromal-immune microenvironment, pleading for a more tailor-made, subtype-based therapeutic approach.^3^^,^^4^

Enhanced inflammatory stromal immune infiltration has been linked to a more favorable clinical outcome in early-stage CRC.^5^ High intratumoral and peritumoral CD8^+^ cytotoxic T lymphocyte (CTL) and CD4^+^ T helper 1 (Th1) content is limited to the hypermutated microsatellite instable (MSI) subtype of CRC.^3^ Recent clinical trials on programmed cell death 1 (PD1)-based immune checkpoint inhibition have confirmed survival benefit in these patients, exploiting the inflammatory immune phenotype, granting Food and Drug Administration approval for the treatment of metastatic mismatch repair–deficient and MSI-high (dMMR-MSI-H) CRC.^6^, ^7^, ^8^, ^9^ In contrast, mismatch repair–proficient and MSI-low (pMMR-MSI-L) CRC, comprising 85% of the total CRC population, has hardly shown any response to immune checkpoint blockade (ICB).^6^^,^^7^^,^^10^ Its impaired clinical activity is hypothesized to be caused by poor immune cell infiltration, decreased inhibitory ligand expression, local immune suppression, and enhanced exhaustion of tumor-infiltrating CTL.^3^^,^^11^^,^^12^ Therefore, there is a need for effective immunotherapy that boosts the impaired tumor immune microenvironment in pMMR CRC.

Promoting anti-tumor immune responses via agonistic targeting of co-stimulatory receptors on tumor-infiltrating lymphocytes (TIL) is a promising alternative to current ICB therapies. In addition to T-cell receptor (TCR) signaling, co-stimulation is required to initiate effective T-cell activation, subset differentiation, effector function, and survival.^13^ Among co-stimulatory receptors, members of the tumor necrosis factor receptor superfamily (TNFRSF) (CD30, DR3, GITR, HVEM, OX-40, TNFR3, 4-1BB) have been widely studied for their application in anti-cancer immunotherapy.^14^^,^^15^ GITR is considered one of the most notable TNFRSF checkpoints because its activation has been shown to activate effector T cells, hamper regulatory T cell (Treg) functionality, and induce efficient anti-tumor responses in preclinical models using agonistic antibodies.^16^, ^17^, ^18^, ^19^, ^20^, ^21^, ^22^, ^23^ Recent phase 1 clinical trials have reported manageable safety profiles for GITR targeting therapy among multiple advanced solid tumors; therefore, this approach may also be relevant for patients with pMMR CRC.^24^, ^25^, ^26^, ^27^, ^28^ Yet, expression and functionality of co-stimulatory checkpoint receptors including GITR on TIL isolated from CRC have not been elucidated.

In this study, we aimed to analyze the expression of TNFRSF members GITR, OX40, and 4-1BB on TIL in pMMR CRC and pMMR CRC-derived liver metastasis (CRLM) patients. Because GITR was expressed most prominently on CD4^+^ and CD8^+^ TIL, in functional assays we focused on co-stimulatory receptor targeting via agonistic GITR ligation in pMMR CRC and CRLM patients. We demonstrate that GITR is expressed by activated CD4^+^ and non-terminally exhausted CD8^+^ TIL. Moreover, GITR co-stimulation is found to be an effective immunomodulatory approach enhancing proliferation and function of TIL from pMMR CRC and CRLM patients. Last, GITR co-stimulation enhanced anti-PD1-mediated immune stimulation in pMMR CRC-derived TIL.

## Results

### Patients

In Tables 1 and 2, an overview of patient characteristics is displayed. One hundred thirty-two tumors of pMMR CRC and CRLM patients were included between July 2016 and July 2022.

**Table 1:** MSS CRC Patient Characteristics

	CRC (N = 95*)*
Age, *y* (interquartile range)	64.9 (58.9–73.7)
Male (*%*)	67 (70.5)
Primary tumor site (*%*)	
Cecum/ascending colon	36 (37.9)
Transverse/descending colon	40 (42.1)
Rectum	19 (20.0)
Pathologic disease stage (*%*)	
I	18 (18.9)
II	35 (36.8)
III	39 (41.1)
IV	3 (3.2)
Histologic subtype (*%*)	
Adenocarcinoma	86 (90.5)
Mucinous carcinoma	8 (8.4)
Other	1 (1.1)
Pretreatment (*%*)	17 (17.9)
Chemotherapy	11 (64.7)
Radiotherapy	8 (47.1)
Other	1 (5.9)

NOTE. Pathologic staging was performed according to American Joint Committee on Cancer, 8^th^ edition: colorectal cancer.

**Table 2:** MSS CRLM Patient Characteristics

	CRLM (N = 37)
Age, *y* (interquartile range)	69.1 (62.7–75.3)
Male (*%*)	23 (62.2)
Primary tumor site (*%*)	
Cecum/ascending colon	8 (21.6)
Transverse/descending colon	17 (46.0)
Rectum	12 (32.4)
Histologic subtype (*%*)	
Adenocarcinoma	37 (100.0)
Mucinous carcinoma	—
Other	—
Pretreatment (*%*)	15 (40.5)
Chemotherapy	12 (80.0)
Radiotherapy	1 (6.7)
Other	3 (20.0)

### GITR Is Predominantly Expressed on Intratumoral CD4^+^ Activated Th and Treg Cells From pMMR CRC and CRLM

**Figure 1:**
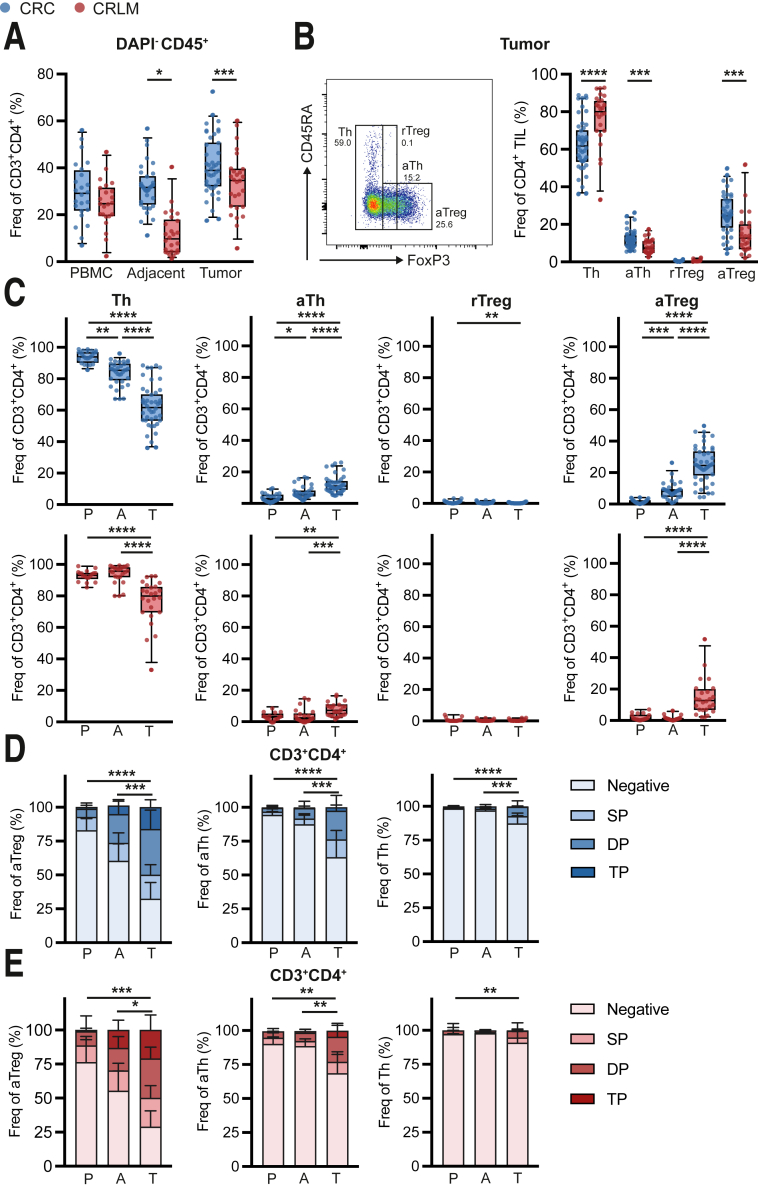
**TNFRSF members are predominantly expressed on intratumoral CD4**^**+**^**activated Th and Treg cells.** (*A*) Frequencies of CD3^+^CD4^+^ among living CD45^+^ cells in tumor, adjacent tissues, and PBMC. CRC are depicted in *blue* (n = 43), and CRLM are depicted in *red* (n = 26). (*B*) Gating strategy and frequencies of Th, aTh, rTreg, and aTreg among CD3^+^CD4^+^ cells in tumor. CRC are depicted in *blue* (n = 42), and CRLM are depicted in *red* (n = 23). (*C*) Frequencies of Th, aTh, rTreg, and aTreg among CD3^+^CD4^+^ cells in tumor (T), adjacent tissues (A), and PBMC (P). CRC are depicted in *blue* (n = 42), and CRLM are depicted in *red* (n = 24). (*D* and *E*, respectively) Frequencies of any TNFRSF member (GITR, 4-1BB, or OX40) negative, single positive (SP), double positive (DP), or triple positive (TP) on aTreg, aTh, and Th in tumor (T), adjacent tissues (A), and PBMC (P). CRC are depicted in *blue* (n = 18), and CRLM are depicted in *red* (n = 9). Friedman (*B*) or Kruskal-Wallis test (*A, C, D,* and *E*) was applied to analyze differences between more than 2 different groups. ∗*P* ≤ .05, ∗∗*P* ≤ .01, ∗∗∗*P* ≤ .001, ∗∗∗∗*P* ≤ .0001. *Boxes and whiskers* represent mean and 95% confidence interval. A, adjacent tissues; aTh, activated T helper; aTreg, activated regulatory T cell; CRC, primary colorectal cancer; CRLM, liver metastasis; DP, double positive; MFI, median fluorescence intensity; P or PBMC, peripheral blood mononuclear cell; rTreg, resting regulatory T cell; SP, single positive; T, tumor; Th, T helper; TIL, tumor-infiltrating lymphocyte; TP, triple positive.

Because we have shown previously that GITR is enriched in CD4^+^ TIL from the hepatic microenvironment, we first focused on CRC- and CRLM-derived CD4^+^ T cells to investigate TNFSRF member expression.^29^^,^^30^ Total CD45^+^ TIL fractions contained more CD4^+^ T cells compared with adjacent tissues. Compared with liver tissues, colorectal tissues demonstrated slightly higher frequencies of CD4^+^ T cells among total CD45^+^ T cells (Figure 1*A*). Effector and regulatory CD4^+^ T-cell subsets were distinguished on the basis of Forkhead box P3 (FoxP3) and CD45RA expression, characterizing Th (CD45RA^+/-^ FoxP3^-^), activated Th (aTh) (CD45RA^-^ FoxP3^low^), resting Treg (rTreg) (CD45RA^+^ FoxP3^low^), and activated Treg (aTreg) (CD45RA^-^ FoxP3^high^) (Figure 1*B*).^31^ CRC and CRLM demonstrated higher proportions of aTh and aTreg in CD4^+^ TIL compared with CD4^+^ T cells in peripheral blood mononuclear cells (PBMC) and adjacent tissues (Figure 1*C*). rTreg were hardly detected in any of the tissue fractions (Figure 1*C*). Compared with CRLM, CRC-derived TIL contained enhanced aTh and aTreg fractions (Figure 1*B*).

To evaluate the potential of co-stimulatory receptor targeting in anti-tumor immunotherapy, TNFSRF member (GITR, 4-1BB, and/or OX40) expression was measured ex vivo on CD4^+^ T cells from pMMR CRC and CRLM patients. Tumor-derived aTreg, aTh, and Th demonstrated higher co-stimulatory receptor expression compared with PBMC and adjacent tissues in CRC (Figure 1*D*). In CRLM, co-stimulatory receptor expression was enhanced on tumor-derived aTreg and aTh compared with PBMC and adjacent tissues (Figure 1*E*).

**Figure 2:**
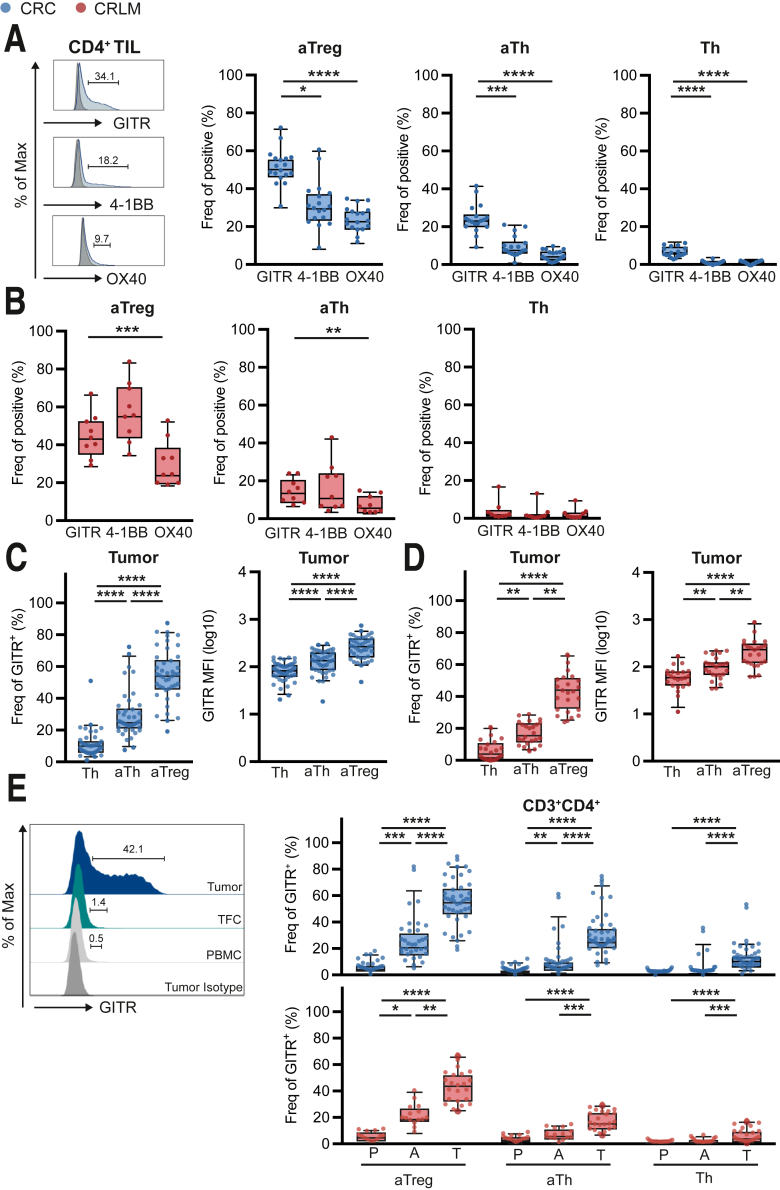
**GITR is predominantly expressed on intratumoral CD4**^**+**^**activated Th and Treg cells.** (*A* and *B*) Frequencies of GITR, 4-1BB, and OX40 on aTreg, aTh, and Th in tumor. CRC are depicted in *blue* (*A*) (n = 18), and CRLM are depicted in *red* (*B*) (n = 9). (*C* and *D*) Frequencies and MFI of GITR on aTreg, aTh, and Th in tumor. CRC are depicted in *blue* (*C*) (n = 42), and CRLM are depicted in *red* (*D*) (n = 23). (*E*) Histogram and frequencies of GITR-positive cells among aTreg, aTh, and Th in tumor (T), adjacent tissues (A), and PBMC (P). CRC are depicted in *blue* (n = 40), and CRLM are depicted in *red* (n = 22). Friedman (*A–D*) or Kruskal-Wallis test (*E*) was applied to analyze differences between more than 2 different groups. ∗*P* ≤ .05, ∗∗*P* ≤ .01, ∗∗∗*P* ≤ .001, ∗∗∗∗*P* ≤ .0001. *Boxes and whiskers* represent mean and 95% confidence interval. A, adjacent tissues; CRC, primary colorectal cancer; CRLM, liver metastasis; MFI, median fluorescence intensity; P or PBMC, peripheral blood mononuclear cell; T, tumor; TIL, tumor-infiltrating lymphocyte.

We compared expression of GITR, 4-1BB, and OX40 on tumor-derived aTreg, aTh, and Th. In CRC, aTreg, aTh, and Th demonstrated enhanced GITR expression compared with 4-1BB and OX40 (Figure 2*A*). Moreover, GITR was expressed more prominently in CRLM-derived aTh and aTreg compared with OX40 (Figure 2*B*). We conclude that high rates of co-expression among TNFSRF members were observed on all CD4^+^ TIL subsets, with GITR being expressed most prominently, especially in CRC.

Therefore, we focused further on GITR expression among CD4^+^ Th, aTh, and aTreg in TIL, adjacent tissues, and PBMC. In CRC and CRLM-derived TIL, GITR was expressed on Th (11.2% ± 1.3% and 5.8% ± 1.1%, respectively) and aTh (29.4% ± 2.4% and 16.9% ± 1.4%, respectively), whereas the highest expression was found on aTreg (54.5% ± 2.5% and 42.8% ± 2.5%, respectively) (Figure 2*C* and *D*). A similar trend was observed with regard to median fluorescent intensities (MFIs) of GITR (Figure 2*C* and *D*).

**Figure 3:**
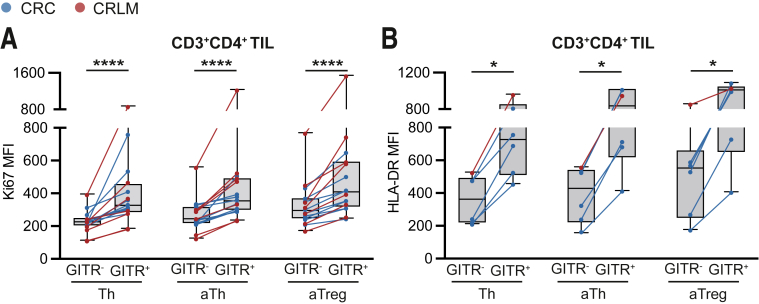
**Intratumoral GITR-expressing Th, aTh, and aTreg feature an activated phenotype.** (*A* and *B*) MFI of proliferation/activation markers Ki67 (*A*) (n = 16) and HLA-DR (*B*) (n = 6) on GITR^-^ and GITR^+^ cells among Th, aTh, and aTreg in tumor. CRC are depicted in *blue*, and CRLM are depicted in *red*. Wilcoxon matched test was used to analyze differences between 2 paired groups. ∗*P* ≤ .05, ∗∗∗∗*P* ≤ .0001. *Boxes and whiskers* represent mean and 95% confidence interval. CRC, primary colorectal cancer; CRLM, liver metastasis; MFI, median fluorescence intensity; TIL, tumor-infiltrating lymphocyte.

Compared with PBMC and adjacent tissues, GITR expression was significantly increased in all CD4^+^ TIL subsets. For CRC Th, expression was increased 0.7% ± 0.2% and 3.0% ± 1.2% vs 11.2% ± 1.3%, respectively; for aTh it was increased 2.5% ± 0.5% and 9.8% ± 2.2% vs 29.4% ± 2.4%, respectively; and for aTreg it was increased 5.1% ± 0.8% and 24.3% ± 2.8% vs 54.5% ± 2.5%, respectively (Figure 2*E*). For CRLM Th, expression was increased 0.7% ± 0.2% and 1.4% ± 0.4% vs 5.8% ± 1.1%, respectively; for aTh it was increased 3.3% ± 0.6% and 6.3% ± 1.1% vs 16.9% ± 1.4%, respectively; and for aTreg it was increased 5.0% ± 0.9% and 20.7% ± 2.1% vs 42.8% ± 2.5%, respectively (Figure 2*E*). Importantly, we observed co-expression of GITR with proliferation and activation markers Ki67 or HLA-DR among all distinct CD4^+^ TIL subsets (Figure 3).

Taken together, we demonstrate that compared with adjacent tissues, expression of the co-stimulatory molecule GITR is increased most prominently of all TNFRSF members investigated on aTh and aTreg in TIL from pMMR CRC and CRLM patients and that its expression is associated with proliferation and activation markers.

### GITR Delineates Activated CD103^+^ CD39^+^ CD8^+^ TIL From pMMR CRC and CRLM

**Figure 4:**
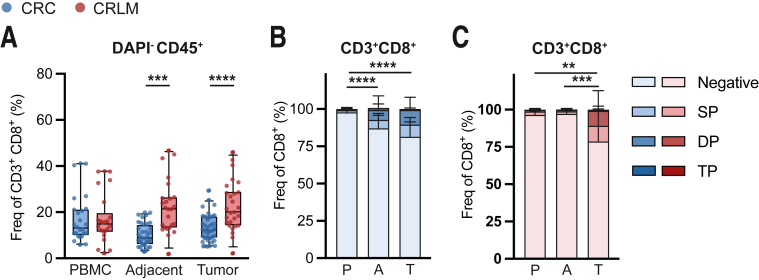
I**ntratumoral CD8 cells are enriched for GITR.** (*A*) Frequencies of CD3^+^CD8^+^ among living CD45^+^ cells in tumor, adjacent tissues, and PBMC. CRC are depicted in *blue* (n = 43), and CRLM are depicted in *red* (n = 26). (*B* and *C*) Frequencies of any TNFRSF member (GITR, 4-1BB, or OX40) negative, single positive (SP), double positive (DP), or triple positive (TP) on CD8^+^ T cells in tumor. CRC are depicted in *blue* (*B*) (n = 18), and CRLM are depicted in *red* (*C*) (n = 9). Mann-Whitney test (*A*) was used to analyze differences between 2 unpaired groups. Kruskal-Wallis test (*B* and *C*) was applied to analyze differences between more than 2 different groups. ∗*P* ≤ .05, ∗∗*P* ≤ .01, ∗∗∗*P* ≤ .001, ∗∗∗∗*P* ≤ .0001. *Boxes and whiskers* represent mean and 95% confidence interval. A, adjacent tissues; CRC, primary colorectal cancer; CRLM, liver metastasis; DP, double positive; P or PBMC, peripheral blood mononuclear cell; SP, single positive; T, tumor.

Subsequently, we studied co-stimulatory molecule expression on cytotoxic CD8^+^ T cells from pMMR CRC and CRLM patients. Compared with colorectal tissues, CRLM demonstrated higher frequencies of CD8^+^ T cells among total CD45^+^ T cells (Figure 4*A*). CD8^+^ T cells were increased in total CD45^+^ TIL fractions compared with adjacent tissues in CRC but not in CRLM.

Tumor-derived CD8^+^ T cells demonstrated higher co-stimulatory receptor expression (GITR, 4-1BB, and/or OX40) compared with PBMC in CRC (Figure 4*B*). In CRLM, co-stimulatory receptor expression was enhanced on tumor-derived CD8^+^ T cells compared with PBMC and adjacent tissues (Figure 4*C*).

**Figure 5:**
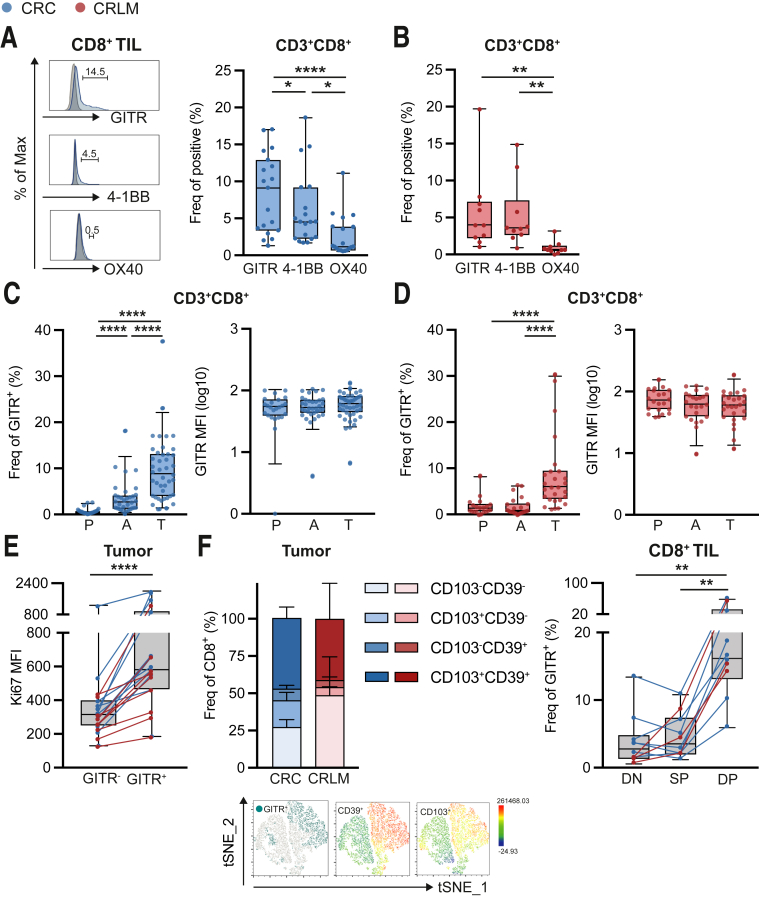
**GITR delineates activated CD103**^**+**^**CD39**^**+**^**CD8**^**+**^**TIL.** (*A* and *B*) Frequencies of GITR, 4-1BB, and OX40 on CD8^+^ T cells in tumor. CRC are depicted in *blue* (*A*) (n = 19), and CRLM are depicted in *red* (B) (n = 10). (*C* and *D*) Frequencies and MFI of GITR on CD8^+^ T cells in tumor (T), adjacent tissues (A), and PBMC (P). CRC are depicted in *blue* (*C*) (n = 42), and CRLM are depicted in *red* (*D*) (n = 23). (*E*) MFI of proliferation marker Ki67 on GITR^-^ and GITR^+^ cells among CD8+ TIL (n = 19). CRC are depicted in *blue*, and CRLM are depicted in *red*. (*F*) Frequencies of CD103^+^CD39^+^, CD103^-^CD39^+^, CD103^+^CD39^-^, and CD103^-^CD39^-^ among CD8^+^ T cells in tumor. *Blue and red bars* represent CRC- and CRLM-derived TIL, respectively (n = 9). tSNE plot demonstrating CD103^+^ and CD39^+^ cells among GITR^+^ CD8^+^ T cells in tumor. Frequencies of GITR-positive cells among DN (CD103^-^CD39^-^), SP (CD103^+^CD39^-^), and DP (CD103^+^CD39^+^) CD8^+^ T cells in tumor (n = 10). Wilcoxon matched test (*E*) was used to analyze differences between 2 paired groups. Friedman (*A*, *B*, and *E*) or Kruskal-Wallis test (*C* and *D*) was applied to analyze differences between more than 2 different groups. ∗*P* ≤ .05, ∗∗*P* ≤ .01, ∗∗∗*P* ≤ .001, ∗∗∗∗*P* ≤ .0001. *Boxes and whiskers* represent mean and 95% confidence interval. A, adjacent tissues; CRC, primary colorectal cancer; CRLM, liver metastasis; DP, double positive; MFI, median fluorescence intensity; P, peripheral blood mononuclear cell; SP, single positive; T, tumor; TIL, tumor-infiltrating lymphocyte.

From the TNFSRF members analyzed in CRC, GITR was expressed at higher frequencies compared with 4-1BB and OX40 on CD8^+^ TIL (Figure 5*A*). On CRLM-derived CD8^+^ TIL, GITR and 4-1BB showed enhanced expression compared with OX40 (Figure 5*B*).

Compared with PBMC and adjacent tissues, GITR expression was increased on CD8^+^ TIL from CRC (9.7% ± 1.1% vs 0.6% ± 0.1% and 3.6% ± 0.6%, respectively) and CRLM (8.4% ± 1.5% vs 1.8% ± 0.4% and 1.6% ± 0.3%, respectively) (Figure 5*C* and *D*). Mean frequencies of GITR expression on CD8^+^ TIL were similar for CRC and CRLM (9.7% and 8.4%, respectively). GITR MFI of CD8^+^ T cells in CRC and CRLM was similar among all fractions (Figure 5*C* and *D*).

Comparison between GITR^+^ and GITR^-^ CD8^+^ TIL revealed co-expression of GITR with proliferation marker Ki67 on CD8^+^ TIL (Figure 5*E*). Recently, Duhen et al^32^ have shown that co-expression of CD39 and CD103 identifies tumor-reactive CD8 T cells in human CRC tumors. In our cohort, we confirmed enrichment of CD39^+^ CD103^+^ (double positive) CD8^+^ T cells among TIL fractions compared with adjacent tissues and PBMC (data not shown). CRC- and CRLM-derived CD8^+^ TIL generally contained 47.5% and 41.1% of CD39^+^ CD103^+^ cells, respectively. Strikingly, CD39^+^ CD103^+^ CD8^+^ TIL appeared to be enriched for GITR (Figure 5*F*).

### GITR Expressing PD1^hi^ CD8^+^ TIL Feature an Exhausted Phenotype With Enhanced Proliferative Capacity in pMMR CRC and CRLM

Because GITR expression on CD8^+^ TIL appears to be linked to a more activated, potentially tumor-reactive phenotype, we aimed to analyze the functional state of GITR^+^ CD8^+^ TIL.

**Figure 6:**
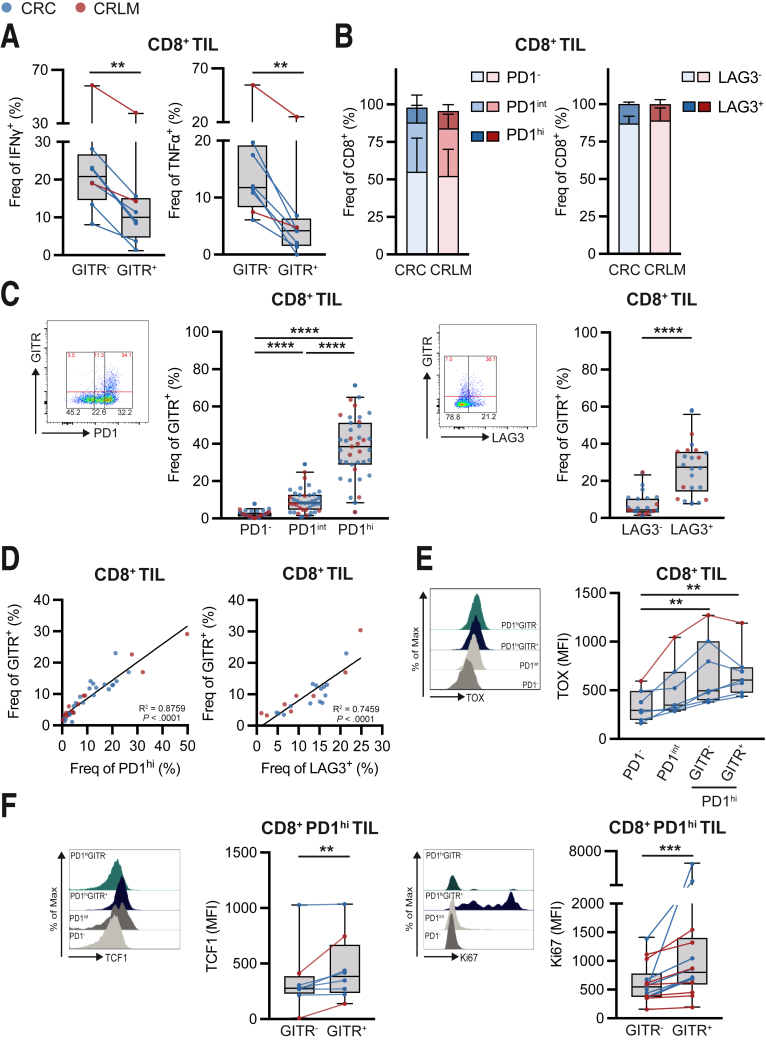
**GITR is up-regulated on PD1**^**hi**^**CD8**^**+**^**TIL featuring a (pre-)exhausted phenotype.** TIL were isolated from tumor tissues using enzymatic digestion and subsequent Ficoll density gradient centrifugation. Ex vivo function and phenotype of TIL were analyzed using flow cytometry with or without in vitro stimulation. (*A*) Frequencies of IFN-γ and TNF-α positive cells in GITR^-^ and GITR^+^ among CD8^+^ T cells in tumor after 5 hours of PMA/ionomycin stimulation (n = 8). CRC are depicted in *blue*, and CRLM are depicted in *red*. (*B*) Frequencies of PD1^-^, PD1^int^, or PD1^hi^ and LAG3^-^ or LAG3^+^ among CD8^+^ T cells in tumor. *Blue and red bars* represent CRC-and CRLM-derived TIL, respectively. (*C*) Gating strategy and frequencies of GITR, PD1, and LAG3 among CD8^+^ T cells in tumor. Frequencies of GITR among PD1^-^, PD1^int^, or PD1^hi^ and LAG3^-^ or LAG3^+^ CD8^+^ T cells in tumor. CRC are depicted in *blue* (n = 26), and CRLM are depicted in *red* (n = 13). (*D*) Correlation of frequencies of GITR-positive cells to frequencies of PD1^hi^ or LAG3^+^ cells among CD8^+^ T cells in tumor. *Blue and red dots* represent individual CRC (n = 26 and n = 14, respectively) and CRLM (n = 13 and n = 8, respectively) patients. (*E*) Histogram and MFI of transcription factor TOX on PD1^-^, PD1^int^, GITR^-^ PD1^hi^, or GITR^+^ PD1^hi^ CD8^+^ T cells in tumor (n = 7). (*F*) Histogram and MFI of TCF1 on GITR^-^ PD1^hi^ or GITR^+^ PD1^hi^ CD8^+^ T cells in tumor (n = 8). Histogram and MFI of proliferation marker Ki67 on GITR^-^ PD1^hi^ or GITR^+^ PD1^hi^ CD8^+^ T cells in tumor (n = 14). CRC are depicted in *blue,* and CRLM are depicted in *red*. Wilcoxon matched test (*A* and *F*) was used to analyze differences between 2 paired groups. Friedman test (*C* and *D*) was applied to analyze differences between more than 2 different groups. Correlation analysis was performed according to Spearman. ∗∗*P* ≤ .01, ∗∗∗*P* ≤ .001, ∗∗∗∗*P* ≤ .0001. *Boxes and whiskers* represent mean and 95% confidence interval. CRC, primary colorectal cancer; CRLM, liver metastasis; IFN-γ, interferon gamma; MFI, median fluorescence intensity; TIL, tumor-infiltrating lymphocyte; TNF-α, tumor necrosis factor alpha.

Although enhanced activation marker expression suggests increased functionality of GITR^+^ CD8^+^ TIL, upon ex vivo restimulation we observed that interferon gamma (IFN-γ) and tumor necrosis factor alpha (TNF-α) secretion by GITR^+^ CD8^+^ TIL were significantly impaired compared with the GITR^-^ counterpart, suggesting a more functionally exhausted state of the GITR^+^ population (Figure 6*A*).

Therefore, we correlated GITR expression with well-defined CD8^+^ T-cell exhaustion markers PD1 and lymphocyte-activation gene 3 (LAG3). Overall, patient-derived TIL from CRC and CRLM contained generally 9.9% and 11.5% PD1^hi^ and 13.0% and 11.0% LAG3^+^ CD8^+^ cells, respectively (Figure 6*B*). We could demonstrate that GITR^+^ CD8^+^ cells were generally concentrated among the PD1^hi^ CD8^+^ TIL and LAG3^+^ CD8^+^ TIL (Figure 6*C*). GITR^+^ CD8^+^ cell frequencies correlated positively with both the frequency of CD8^+^ PD1^hi^ and LAG3^+^ TIL (R^2^ = 0.86 and 0.75, respectively; Figure 6*D*). Furthermore, transcription factor TOX was demonstrated to be up-regulated by PD1^hi^ CD8^+^ TIL independently of GITR expression (Figure 6*E*). However, remarkably, TCF1 expression was enhanced in PD1^hi^ GITR^+^ CD8^+^ TIL compared with PD1^hi^ GITR^-^ cells, and the former population showed significantly enhanced Ki67 expression (Figure 6*F*).

These data demonstrate that GITR is predominantly expressed by functionally impaired CD8^+^ TIL characterized by enhanced inhibitory receptor and TOX expression ex vivo. Nevertheless, GITR^+^ PD1^hi^ CD8^+^ TIL differ from GITR^-^ PD1^hi^ CD8^+^ TIL because they demonstrate an enhanced proliferative capacity based on enhanced Ki67 and TCF1 expression. Agonistic targeting of GITR might potentially functionally reinvigorate this cell population.

### GITR Ligation Enhances pMMR CRC- and CRLM-Derived CD4^+^/CD8^+^ TIL Expansion and Pro-inflammatory Cytokine Secretion

**Figure 7:**
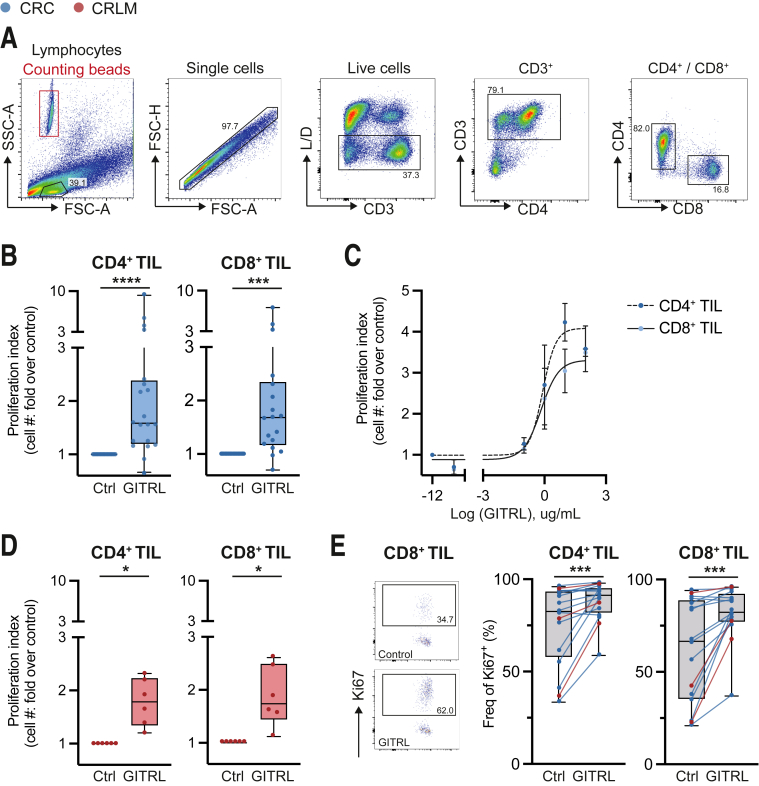
**GITR ligation enhances CD4**^**+**^**and CD8**^**+**^**TIL expansion**. TIL were isolated from tumor tissues using enzymatic digestion and subsequent Ficoll density gradient centrifugation. TIL were stimulated using CD3/CD28 stimulation beads in the absence (ctrl) or presence of a hexameric GITR ligand (GITLR 1 μg/mL + anti-HA 2.5 μg/mL). After 8 days, TIL expansion was measured and defined as fold increase over control conditions (proliferation index). (*A*) Flow cytometric gating strategy used for ex vivo phenotyping after in vitro polyclonal assay. Cell numbers were normalized using counting beads (*red box*). (*B*) pMMR CRC-derived CD4^+^ and CD8^+^ TIL expansion upon GITR ligation (n = 19). (*C*) CD4^+^ and CD8^+^ TIL expansion upon various dosages of GITR ligation (0.001 μg/mL, 0.01 μg/mL, 0.1 μg/mL, 1.0 μg/mL, 10.0 μg/mL, and 100.0 μg/mL, respectively) (n = 8). (*D*) pMMR CRLM-derived CD4^+^ and CD8^+^ TIL expansion upon GITR ligation (n = 6). (*E*) Gating strategy and frequencies of Ki67^+^ cells among CD4^+^ and CD8^+^ TIL upon GITR ligation, respectively. CRC are depicted in *blue*, and CRLM are depicted in *red*. Wilcoxon matched test (*B, D,* and *E*) was used to analyze differences between 2 paired culture conditions. Friedman test (*C*) was applied to analyze differences between more than 2 different culture conditions. ∗*P* ≤ .05, ∗∗∗*P* ≤ .001, ∗∗∗∗*P* ≤ .0001. *Boxes and whiskers* represent mean and 95% confidence interval. CRC, primary colorectal cancer; CRLM, liver metastasis; Ctrl, control; TIL, tumor-infiltrating lymphocyte.

The immune modulatory effect of GITR ligation on TIL was tested in vitro. Primary TIL were cultured in the presence of anti-CD3/-CD28 activation beads providing initial TCR and CD28 signaling, which is required for efficient GITR-mediated co-stimulation. After 8–10 days of in vitro culture, CD4^+^ and CD8^+^ TIL expansion was determined by ratiometric evaluation of cell numbers by flow cytometry using counting beads (Figure 7*A*).

Variable baseline proliferation rates of TIL were observed among individual patients, but overall co-culture in the presence of hexameric GITRL doubled the numbers of CD4^+^ and CD8^+^ pMMR CRC- and CRLM-derived TIL compared with TIL stimulated in the presence of αCD3/CD28 alone (Figure 7*B*–*D*). We could demonstrate a dose-dependent increase for both CD4^+^ and CD8^+^ TIL proliferation (Figure 7*C*). Moreover, proliferation marker Ki67 was significantly enhanced upon GITRL treatment for both TIL subsets (Figure 7*E*). Correlating with the ex vivo GITR expression data, CD4^+^ TIL demonstrated higher Ki67 frequencies compared with CD8^+^ TIL upon GITR ligation. Nevertheless, expansion rates did not significantly differ between both TIL subsets (data not shown).

**Figure 8:**
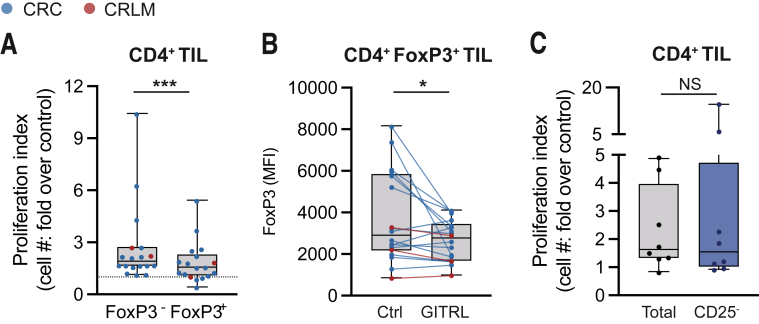
**CD4**^**+**^**Th TIL respond to GITR ligation in the absence of CD4**^**+**^**Treg**. TIL were isolated from tumor tissues using enzymatic digestion and subsequent Ficoll density gradient centrifugation. TIL were stimulated using CD3/CD28 stimulation beads in the absence (ctrl) or presence of hexameric GITR ligand (GITRL 1 μg/mL + anti-HA 2.5 μg/mL) with or without magnetic CD25-depletion. TIL expansion was measured and defined as fold increase over control conditions (proliferation index). (*A*) pMMR CRC- and CRLM-derived CD4^+^ FoxP3^-^ and CD4^+^ FoxP3^+^ TIL expansion upon GITR ligation (n = 18). (*B*) MFI of FoxP3 in pMMR CRC- and CRLM-derived CD4^+^ FoxP3^+^ TIL in absence (ctrl) or presence of hexameric GITR ligand (n = 18). (*C*) pMMR CRC-derived CD4^+^ and CD8^+^ TIL expansion upon GITR ligation with or without CD25-depletion. (n = 8). Wilcoxon matched test was used to analyze differences between 2 paired culture conditions. ∗*P* ≤ .05, ∗∗*P* ≤ .01. *Boxes and whiskers* represent mean and 95% confidence interval. CRC, primary colorectal cancer; CRLM, liver metastasis; MFI, median fluorescence intensity; TIL, tumor-infiltrating lymphocyte.

To study how GITR ligation affects different CD4^+^ TIL subsets, we compared the expansion of FoxP3^-^ and FoxP3^+^ CD4^+^ TIL in vitro. Generally, after GITR ligation FoxP3^-^ CD4^+^ TIL demonstrated enhanced expansion compared with FoxP3^+^ TIL (Figure 8*A*). Furthermore, FoxP3 MFI declined upon GITR ligation, suggesting a reduced immune suppressive capacity, as was described previously in liver cancers (Figure 8*B*).^21^ To confirm the ability of CD4^+^ Th TIL to respond to GITR ligation independently of Treg, we cultured CD25-depleted TIL for 8–10 days. CD4^+^ TIL demonstrated similar expansion rates compared with controls in the presence or absence of CD25^+^ CD4^+^ Treg TIL, suggesting that the presence of aTreg within the TIL does not negatively impact the effect of GITR ligation on T-cell proliferation (Figure 8*C*).

**Figure 9:**
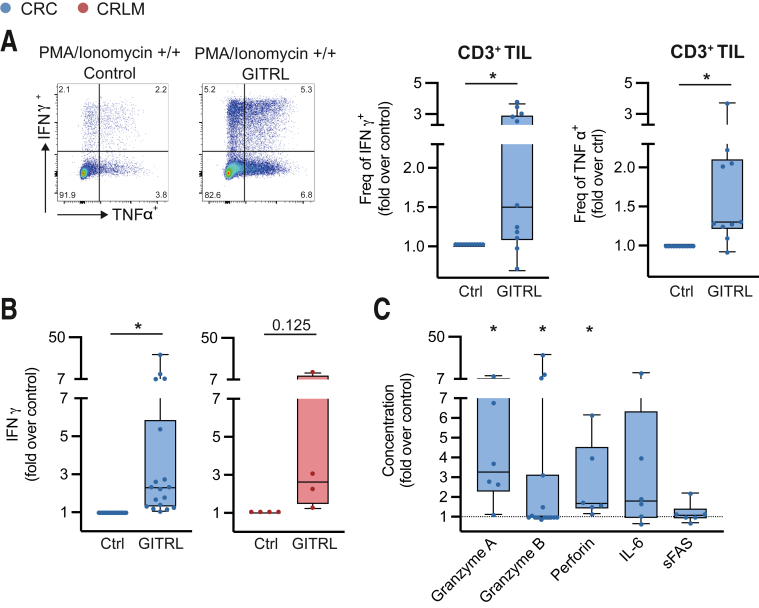
**GITR ligation increases CD3**^**+**^**TIL pro-inflammatory cytokine secretion**. TIL were isolated from tumor tissues using enzymatic digestion and subsequent Ficoll density gradient centrifugation. TIL were stimulated using CD3/CD28 stimulation beads in absence (ctrl) or presence of hexameric GITR ligand (GITRL 1 μg/mL + anti-HA 2.5 μg/mL). After 8 days, supernatants were analyzed for soluble factors, and TIL were phenotyped after PMA/ionomycin restimulation. (*A*) Flow cytometric gating strategy and relative frequencies of IFN-γ and TNF-α producing pMMR CRC-derived CD3^+^ TIL upon PMA/ionomycin restimulation after GITR ligation compared with control conditions (n = 11). (*B*) Concentration of secreted IFN-γ defined as fold increase over control conditions. CRC are depicted in *blue* (n = 18), and CRLM are depicted in *red* (n = 4). (*C*) Concentration of secreted cytokines defined as fold increase over control conditions. CRC are depicted in *blue* (n = 6). Wilcoxon matched test was used to analyze differences between 2 paired culture conditions. ∗*P* ≤ .05. *Boxes and whiskers* represent mean and 95% confidence interval. CRC, primary colorectal cancer; CRLM, liver metastasis; Ctrl, control; IFN-γ, interferon gamma; TIL, tumor-infiltrating lymphocyte; TNF-α, tumor necrosis factor alpha.

To evaluate pro-inflammatory effector cytokine secretion by CD3^+^ TIL, after 8–10 days of in vitro culture with or without GITR ligation, total TIL were restimulated using PMA-ionomycin. Upon restimulation, CD3^+^ TIL that were treated with GITRL demonstrated increased frequencies of both IFN-γ^+^ and TNF-α^+^ cells compared with non-treated TIL (Figure 9*A*). These data were confirmed by the detection of elevated IFN-γ levels in the culture supernatants of pMMR CRC and CRLM TIL that were treated with GITR ligation (Figure 9*B*). Furthermore, pro-inflammatory proteases granzyme A (GzmA), granzyme B (GzmB), and perforin were increased in response to GITRL in CRC-derived TIL cultures as well (Figure 9*C*).

**Figure 10:**
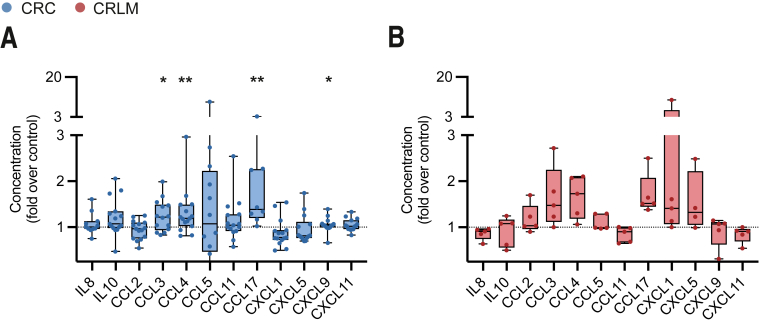
**GITR ligation skews TIL to a pro-inflammatory chemokine profile.** TIL were isolated from tumor tissues using enzymatic digestion and subsequent Ficoll density gradient centrifugation. TIL were stimulated using CD3/CD28 stimulation beads in absence (ctrl) or presence of hexameric GITR ligand (GITRL 1 μg/mL + anti-HA 2.5 μg/mL). After 8 days, supernatants were analyzed for soluble factors. (*A*) Concentration of secreted cytokines and chemokines defined as fold increase over control conditions of CRC-derived TIL (n = 10). (*B*) Concentration of secreted cytokines and chemokines defined as fold increase over control conditions of CRLM-derived TIL (n = 5). Wilcoxon matched test was used to analyze differences between 2 paired culture conditions. ∗*P* ≤ .05, ∗∗*P* ≤ .01. *Boxes and whiskers* represent mean and 95% confidence interval. CRC, primary colorectal cancer; CRLM, liver metastasis; sFAS, soluble FAS; TIL, tumor-infiltrating lymphocyte.

Remarkably, GITR ligation not only skewed TIL to enhance pro-inflammatory cytokine secretion but also improved pro-inflammatory chemokine secretion. In CRC-derived TIL cultures, we detected enhanced CCL3, CCL4, CCL17, and CXCL9 levels (Figure 10*A*). CCL3, CCL4, CCL17, CXCL1, and CXCL5 levels showed a trend to increase among CRLM-derived cultures (Figure 10*B*).

Taken together, these data demonstrate that GITR ligation reinvigorates CRC- and CRLM-derived CD4^+^ and CD8^+^ TIL functionality, leading to enhanced expansion and pro-inflammatory cytokine or chemokine secretion.

### GITR Ligation Potentiates Immune Stimulation of Anti-PD1 in pMMR CRC-derived TIL

So far, PD1 blockade monotherapy has not shown a clinical benefit in pMMR CRC and CRLM patients. Our data demonstrate that tumor-derived GITR^+^ CD8^+^ cells were generally concentrated among the PD1^hi^ CD8^+^ TIL. Therefore, we studied whether co-stimulatory checkpoint targeting via GITR ligation could enhance anti-PD1-mediated T-cell reinvigoration in vitro.

**Figure 11:**
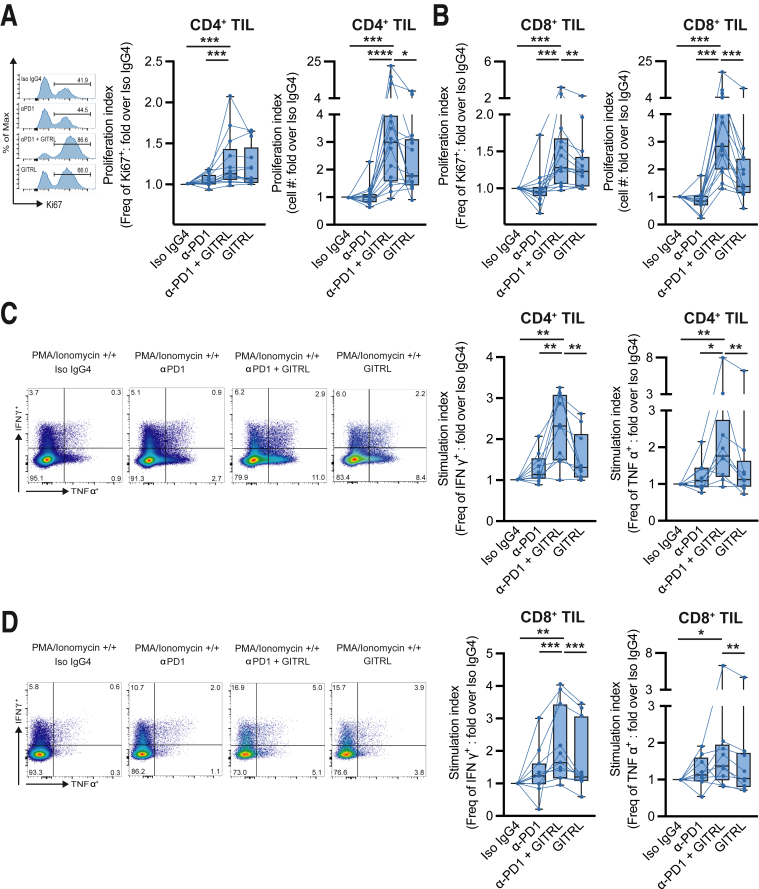
**GITR ligation further enhances anti-PD-1-mediated reinvigoration of CD4**^**+**^**and CD8**^**+**^**TIL.** TIL were isolated from tumor tissues using enzymatic digestion and subsequent Ficoll density gradient centrifugation. TIL were stimulated using CD3/CD28 stimulation beads in presence of isotype antibodies (iso IgG4), anti-PD1 blocking antibodies (∝ PD1), anti-PD1 blocking antibodies in combination with GITRL (∝ PD1 + GITRL), or GITRL (GITRL). After 8 days, TIL were phenotyped directly or after PMA/ionomycin restimulation. TIL expansion and cytokine secretion were measured and defined as fold increase over isotype control conditions (proliferation and stimulation index, respectively). (*A*) pMMR CRC-derived CD4^+^ TIL Ki67 expression and expansion upon iso IgG4, αPD1, ∝ PD1 + GITRL, and GITRL (n = 12). (*B*) pMMR CRC-derived CD8^+^ TIL Ki67 expression and expansion upon iso IgG4, ∝ PD1, ∝ PD1 + GITRL, and GITRL (n = 12). (*C*) Flow cytometric gating strategy and relative frequencies of IFN-γ and TNF-∝ producing pMMR CRC-derived CD4^+^ TIL upon PMA/ionomycin restimulation after ∝ PD1, ∝ PD1 + GITRL, and GITRL compared with isotype control conditions (n = 10). (*D*) Flow cytometric gating strategy and relative frequencies of IFN-γ and TNF-∝ producing pMMR CRC-derived CD8^+^ TIL upon PMA/ionomycin restimulation after ∝ PD1, ∝ PD1 + GITRL, and GITRL compared with isotype control conditions (n = 10). Friedman (*A–D*) was applied to analyze differences between more than 2 different groups. ∗*P* ≤ .05, ∗∗*P* ≤ .01, ∗∗∗*P* ≤ .001, ∗∗∗∗*P* ≤ .0001. *Boxes and whiskers* represent mean and 95% confidence interval. CRC, primary colorectal cancer; IFN-γ, interferon gamma; Iso IgG4, isotype control; PI, proliferation index; SI, stimulation index; TIL, tumor-infiltrating lymphocyte; TNF-∝, tumor necrosis factor alpha.

In the majority of patient-derived TIL, anti-PD1 monotherapy did not enhance TIL expansion or effector cytokine production (Figure 11). However, GITR co-stimulation in combination with PD1 blockade did enhance pMMR CRC-derived CD4^+^ and CD8^+^ expansion (Figure 11*A* and *B*). Conformably, proliferation marker Ki67 was significantly enhanced upon combination treatment for CD8^+^ TIL (Figure 11*B*). Ultimately, IFN-γ production in CD4^+^ and CD8^+^ TIL was enhanced upon PMA-ionomycin restimulation after in vitro culture with the combination regimen as well as TNF-α production in CD4^+^ TIL, indicating that GITR co-stimulation improves anti-PD1-mediated immune stimulation in pMMR CRC-derived TIL (Figure 11*C* and *D*).

## Discussion

Whereas clinical trials on PD1-based immune checkpoint inhibition have shown great anti-tumor efficacy in dMMR (MSI-high) CRC, pMMR (MSI-low or microsatellite stable [MSS]) tumors that occur in the majority of CRC and CRLM patients do not respond well to ICB.^6^^,^^7^^,^^10^ Presently, the implementation of co-stimulatory checkpoint targeting in anti-tumor immunotherapy is widely studied in preclinical and early clinical trials as a suitable alternative or supportive approach for ICB. Yet, the biological and clinical implications of these receptors in anti-tumor immunotherapy remain elusive. In this study, we aimed to comprehensively analyze the expression of TNFRSF members GITR, OX40, and 4-1BB on CD4^+^ and CD8^+^ TIL and to associate their expression to T-cell activation or exhaustion status in pMMR CRC and CRLM patients. Furthermore, we studied the impact of GITR co-stimulation on pMMR-CRC- and -CRLM-derived-TIL functions.

In a cohort of 132 patients with pMMR CRC or CRLM, we first demonstrated that GITR was expressed more prominently on tumor-derived TIL compared with OX40 and 4-1BB. Furthermore, GITR was predominantly co-expressed with OX40 and 4-1BB and compromised the vast majority of TIL expressing a TNFRSF member. GITR expression was enhanced on activated, proliferating CD4^+^ and CD8^+^ TIL. CD4^+^ aTh and aTreg displayed highest GITR expression compared with their counterparts in adjacent tissues and blood. Notably, GITR expression in CD8^+^ TIL delineated putative tumor reactive TIL.

To our knowledge, this is the first comprehensive report of GITR expression in human patient-derived pMMR CRC tissues. Co-stimulatory TNFRSF members have been described to be differentially expressed on TIL from different tumor types. Recently, Kim et al^33^ demonstrated enhanced 4-1BB expression on CD8^+^ TIL from hepatocellular carcinoma (HCC) compared with expression of various other TNFRSF members (eg, GITR, TNFR2, HVEM, DR3, OX40, CD30). Others have demonstrated varying levels of GITR expression in the tumor microenvironment (TME) from several solid tumors (non-small cell lung carcinoma, renal cell carcinoma, melanoma, and HCC). However, in all tumor types GITR expression was highest on CD4^+^ and CD4^+^ FoxP3^+^ TIL.^34^ We demonstrate that also in pMMR CRC and CRLM, compared with other TNFRSF members, GITR is prominently expressed by CD4^+^ TIL, co-expressed with proliferation and activation markers (Ki67 and HLA-DR, respectively), and enriched within the TME compared with adjacent tissues and blood.

Enhanced GITR expression was detected on CD4^+^ CD45RA^-^ FoxP3^hi^ aTreg and non-suppressive CD4^+^ CD45RA^-^ FoxP3^lo^ aTh. Improved activation and anti-tumor immunity via GITR engagement in CD4^+^ TIL are described to be mediated either by CD4^+^ Treg depletion via Fcγ-receptor co-engagement on innate immune cells or CD4^+^ Treg functionality impairment via lineage destabilization.^22^^,^^35^^,^^36^ We have confirmed previously that co-stimulation via GITR could alleviate Treg-mediated immune suppression, restoring proliferative capacity and cytokine production of non-Treg (CD4^+^CD25^-^) TIL from primary and secondary liver tumors.^37^ Nevertheless, multiple studies have shown a direct stimulatory effect of GITR ligation on effector TIL as well.^20^^,^^22^^,^^38^^,^^39^ Moreover, hexameric GITR ligation (GITRL) in human naive PBMC was shown to induce CD4^+^ T-cell proliferation in the absence of Treg.^40^ In CRC, TME-residing aTh have been shown to correlate with improved survival rates because they produce pro-inflammatory cytokines and harbor anti-tumor immunity.^31^^,^^41^ Although on the basis of our in vitro functional polyclonal assay we cannot distinguish between any direct or indirect effects on CD4^+^ (FoxP3^+^) or CD8^+^ TIL, these findings by us and others suggest that immunotherapeutic targeting of GITR may stimulate the local immune response within the CRC-/CRLM-related TME through affecting both Treg and aTh cell function.

In CRC, Duhen et al^32^ and Simoni et al^42^ have demonstrated that CD103^+^CD39^+^ CD8^+^ TIL are enriched by tumor-reactive T cells. Here, we confirmed enrichment of CD103 and CD39 co-expression on CD8^+^ T cells in TIL compared with adjacent tissues and blood (data not shown). Interestingly, GITR expression was highest on CD103^+^CD39^+^ CD8^+^ TIL, suggesting that tumor-reactive CD8^+^ T cells within the TME display GITR expression. Furthermore, enhanced expression of Ki67, HLA-DR, and perforin illustrates the pro-inflammatory state of these putative tumor reactive GITR^+^ CD8^+^ TIL. Yet, ex vivo CD8^+^ TIL stimulation revealed impaired effector cytokine secretion in GITR^+^ cells compared with GITR^-^ TIL, suggesting the former to be functionally impaired. Functional exhaustion of these cells might be caused by potential chronic T-cell receptor stimulation of CD103^+^CD39^+^ CD8^+^ TIL in the TME.^43^ In support of this hypothesis, we observed enrichment of GITR among PD1^hi^ and LAG3^+^ CD8^+^ TIL. Interestingly, although there was enhanced transcription factor TOX expression in PD1^hi^ GITR^-^ and GITR^+^ CD8^+^ TIL, the latter showed enhanced TCF1 and Ki67 expression compared with PD1^hi^ GITR^-^ CD8^+^ TIL. Therefore, GITR seems to delineate PD1^hi^ CD8^+^ TIL into cells that demonstrate some similarity to exhausted T (Tex) precursor cells. In recent preclinical cancer models, these Tex precursor cells have been shown to be a crucial source for providing a robust response to ICB, suggesting that these cells may be eligible for GITR-mediated reactivation as well.^44^^,^^45^

Whereas TNFRSF co-stimulation was thought to affect CD8^+^ TIL indirectly via its selective effect on CD4^+^ TIL, direct effects of GITRL on CD8^+^ TIL have been described.^20^^,^^38^ We performed polyclonal functional assays testing the ability of GITRL to stimulate tumor-derived CD4^+^ and CD8^+^ TIL function ex vivo. We demonstrated enhanced Ki67 expression and expansion of CD4^+^ and CD8^+^ TIL from pMMR CRC and CRLM. In addition, tumor-derived T cells that were treated with GITRL demonstrated increased frequencies of both IFN-γ^+^ and TNF-α^+^ cells and enhanced secretion of pro-inflammatory proteases and chemokines (eg, CCL3, CCL4, CXCL9), potentially driving immune cells attraction and activation in the TME in vivo.^46^

In mice, dual therapy of GITR co-stimulation and PD1 blockade was shown to enhance effector T-cell function synergistically by restoring the balance of key homeostatic regulators CD226 and T-cell immunoreceptor with Ig and ITIM domains.^20^ Moreover, it has been shown recently that the anti-GITR/anti-PD1 combination could restore the effector T-cell:Treg ratio, resulting in enhanced anti-tumor immunity.^25^^,^^39^ In concordance with clinical studies, our data indicate that anti-PD1 monotherapy hardly stimulates the activity of tumor-derived TIL from pMMR CRC in vitro. However, the combination regimen of anti-PD1 with GITRL led to significant functional T-cell reinvigoration. Because high GITR expression is mostly restricted to the tumor site, which potentially limits systemic adverse events of GITR targeting, in our opinion GITR represents an attractive target for therapeutic immunomodulation alone or in combination with ICB in these pMMR patients. Although treatment with anti-GITR antibodies had been shown to cause toxicity upon repeated administration in preclinical animal models, recently it has been shown that a newly developed fully human agonistic GITR immunoglobulin G1 monoclonal antibody in combination with nivolumab demonstrated an acceptable safety profile in patients with advanced solid tumors.^26^^,^^47^

In humans, GITR monoclonal antibodies have been shown to reduce circulating and intratumoral Treg.^25^ Yet, efficient direct T-cell activation via TNFR requires receptor clustering to induce optimal downstream signal transduction.^19^ In our study, GITR oligomerization is facilitated using a hexameric GITRL. Other strategies to induce receptor multimerization are Fc-engineering or the construction of bi-specific antibodies.^48^^,^^49^ Chan et al^49^ have recently shown the great potential of combining immune checkpoint stimulation (ICS) and ICB using an anti-PD-1-GITR-L bispecific. In particular, the approach of bispecific antibodies deserves great attention for development of future clinical trials on ICS because these constructs not only drive receptor multimerization but also direct GITR-mediated activation to tumor reactive PD-1 expressing cells only.

The strength of our study is the use of human patient-derived TIL, but it entails some limitations as well.^1^ Because all patients underwent surgical resection, our study cohort does not comprise the full spectrum of the CRC and CRLM patient population. Currently, the efficacy of immunotherapy has been mostly studied in advanced therapy resistant tumors. Nevertheless, the (neo)adjuvant application of immunotherapy in early or intermediate stage disease is being studied more and more frequently.^2^ Some patients from our CRC and CRLM cohort have undergone systemic therapy before resection. Therapeutic regimen might have varied among individual patients, potentially changing the composition of the TME. To exclude any direct effects of neoadjuvant therapy on the TME, minimum time before surgery was set at 4 weeks.^3^ TIL numbers isolated from individual patient tissues were highly variable. Therefore, phenotype analysis and in vitro functional assays could not be performed in all patients.

In summary, we conclude that GITR is enriched in CD4^+^ aTh and aTreg TIL as well as in PD1^hi^ CD8^+^ TIL. Furthermore, GITRL enhances pMMR CRC- and CRLM-derived human TIL functionality ex vivo. Our study provides compelling preclinical data that support agonistic targeting of GITR as part of a new immunotherapeutic approach for pMMR CRC and CRLM patients.

## Methods

### Patient Selection

Patients (age >18 years) undergoing surgical resection between July 2016 and November 2021 for either primary CRC (stage 1–3) or CRLM were included from 3 different hospitals. Patients having received (any) neoadjuvant treatment or immunosuppressive therapy <4 weeks and <3 months before surgical resection were excluded from our study. Peripheral blood was drawn on the day of the surgical resection before surgery. In accordance with the pathologists, fresh samples of tumor and adjacent tumor-free tissues (>10 cm and >2 cm distant of the tumor for primary CRC and liver metastasis, respectively) were obtained and processed within 24 hours. Patients’ mismatch repair status was determined using immunohistochemistry and defined by the loss of expression of any of the following mismatch repair gene-related proteins: MLH1, PMS2, MSH2, and MSH6. Patient data were retrieved from electronical medical records. All study procedures were approved by the local ethics committee (NL58534.078.16; NL47888.041.14). Patients had given informed consent for tissue and blood donation as well as usage of personal data.

### Cell Preparation

PBMC were isolated by Ficoll density gradient centrifugation. Mononuclear single cell suspensions from tumor and adjacent tissues were obtained by enzymatic tissue digestion. Adjacent colon tissues were cut and stirred in the presence of EDTA (10% fetal calf serum, 15 mmol/L HEPES, 1 mmol/L EDTA in phosphate-buffered saline) 4 times for 15 minutes at 37°C. Subsequently, adjacent colon as well as cut primary CRC tissues were digested in the presence of 400 U/mL collagenase type VIII (Sigma-Aldrich, St Louis, MO) and 0.2 mg/mL DNAse I (Sigma-Aldrich) in Hanks balanced salt solution for 30–60 minutes at 37°C with interrupted gently swirling. CRLM and adjacent liver tissues were cut into small pieces and then digested with 0.2 mg/mL DNAse I (Sigma-Aldrich) and 0.125 mg/mL collagenase type IV (Sigma-Aldrich) for 40 minutes at 37°C with interrupted gently swirling. Cell suspensions were filtered through 70-μm pore cell strainers (BD Biosciences, San Diego, CA), and mononuclear leukocytes were obtained by Ficoll density gradient centrifugation. Mononuclear cell viability, CD45/CD3 purities, and cell numbers were determined using flow cytometry (MACS Quant; Miltenyi Biotec, Bergisch Gladbach, Germany). A minimum of 100,000 total CD3^+^ TIL/1 g tissue was set for study inclusion.

### Flow Cytometric Analysis

Freshly isolated PBMC, tumor-infiltrating lymphocytes, and tissue-infiltrating lymphocytes were analyzed for the expression of specific surface and intracellular markers using monoclonal antibodies (Table 3). Nonviable cells were excluded by labeling with Fixable Viability Dye Efluor 506 (eBioscience, Vienna, Austria). Cell surface staining with fluorochrome-conjugated antibodies was performed in the dark at 4°C for 30 minutes, after which cells were fixed, permeabilized using the FoxP3 staining buffer kit (eBioscience), and stained for intracellular antigens. Cells were measured using a FACSCanto II, FACSAria SORP II, or FACSymphony flow cytometer (BD Biosciences) and analyzed using FlowJo software version V10 (BD Biosciences). Appropriate isotype control antibodies were used for gating purposes (Table 3).

**Table 3:** Antibody List Used for Flow Cytometry

Specificity	Fluorochrome	Clone	Supplier
CD3	APC-R700	UCHT1	BD Biosciences
CD3	SB780	UCHT1	eBioscience
CD3	PerCP-Cy5.5	SK7	BD Biosciences
CD4	APC-Fire750	RPA-T4	Biolegend
CD4	BV605	RPA-T4	BD Biosciences
CD4	APC-eFluor780	OKT4	eBioscience
CD45	PE-CF594	HI30	BD Biosciences
CD45RA	PE-CF594	HI30	BD Biosciences
CD8	APC	RPA-T8	Biolegend
CD8	SB645	OKT8	eBioscience
CD8	PE	RPA-T8	Biolegend
CD8a	APC-Fire750	RPA-T8	Biolegend
CD8a	PerCP-Cy5.5	RPA-T8	eBioscience
CD39	BV421	A1	Biolegend
CD103	PE-Cy7	Ber-ACT8	Biolegend
FoxP3	eFluor450	236A/E7	eBioscience
FoxP3	PE	236A/E7	eBioscience
GITR (CD357)	FITC	22-04-2022	R&D Systems
Granzyme B	V450	GB11	BD Biosciences
HLA-DR	APC	LN3	eBioscience
IFN-γ	FITC	25723.11	BD Biosciences
IFN-γ	PE-Cy7	4S.B3	eBioscience
Isotype mIgG1	APC	MOPC-21	Biolegend
Isotype mIgG1	PE	P3.6.2.8.1	eBioscience
Isotype mIgG1	FITC	P3	eBioscience
Isotype mIgG1	Pe-Cy7	MOPC-21	Biolegend
Isotype mIgG1	BV421	X40	BD Biosciences
Isotype mIgG1	PerCP-Cy5.5	MOPC-21	BD Biosciences
Ki67	APC	20Raj1	eBioscience
LAG3 (CD223)	BV421	T47-530	BD Biosciences
OX40 (CD134)	PE	ACT35	eBioscience
Perforin	APC-Cy7	dG9	Biolegend
PD1 (CD279)	PE	MIH4	eBioscience
PD1 (CD279)	Pe-Cy7	J105	eBioscience
TCF1	PE	7F11A10	Biolegend
TNF-α	PerCP-Cy5.5	MAb11	Biolegend
TNF-α	APC	MAb11	eBioscience
TOX	APC	REA473	Miltenyi
4-1BB (CD137)	APC	4B4-1	BD Biosciences

### Ex Vivo Polyclonal T-Cell Activation Assay

Isolated total TIL were either used directly for in vitro culture or purified using Magnetic Cell Separation. CD25-depleted TIL fractions were obtained by negatively selecting CD25^-^ TIL with human CD25 MicroBeads II (130-0920983; Miltenyi Biotec). Efficiency of Magnetic Cell Separation was set to a minimum of 90% purity of FoxP3- within CD25^-^ TIL fractions. TIL cultures were performed in RPMI 1640 (Lonza, Breda, the Netherlands) supplemented with 10% human AB serum (Sigma-Aldrich), 2 mmol/L L-glutamine (Invitrogen, Waltham, MA), 50 mmol/L Hepes Buffer (Lonza), 1% penicillin-streptomycin (Gibco-Life Technologies, Oslo, Norway), 5 mmol/L sodium pyruvate (Gibco-Life Technologies), and 1% minimum essential medium non-essential amino acids (complete medium) at 37°C and 5% CO_2_. TIL were cultured in a 96-well round-bottom culture plate (0.5 × 10E6 CD45^+^ cells/well) in the presence of anti-human anti-CD3/CD28 Dynabeads (cell:bead ratio 100:1; Gibco-Life Technologies). Experiments in the range of >15% baseline Ki67 expression on T cells after day 8-9 of culture were included unless mentioned otherwise. Overall, suboptimal CD3/CD28-mediated pre-stimulation could be reached in approximately 85% of all vitro TIL cultures. For indicated experiments, cells were treated with 1 μg/mL azide-free and low endotoxin soluble GITRL (R&D Systems, Minneapolis, MN) crosslinked with 2.5 μg/mL anti-HA antibody (R&D Systems) alone or in combination with 10 μg/mL humanized immunoglobulin G4 blocking anti-PD1 antibody (Nivolumab; Bristol-Myers Squibb, New York, NY) or the corresponding isotype control antibody (hIgG4, clone QA16A15; Biolegend, San Diego, CA). After 8–9 days, culture supernatants were collected and quantified for cytokines/chemokines using LegendPlex Human CD8/NK Panel and/or Human Pro-Inflammatory Chemokine Panel (740267 and 740985, respectively; Biolegend). For indicated experiments, cells were restimulated (on day 0 and day 8/9) using PMA (40 ng/mL; Sigma-Aldrich), ionomycin (1 μg/mL; Sigma-Aldrich), and Monensin (1000X; eBioscience). T-cell expansion was determined on the basis of ratiometric determination of absolute cell counts using counting beads (01-1234-42; Thermo Fisher Scientific, Waltham, MA).

### Statistical Analysis

Statistical analyses were performed using GraphPad Prism software version 9.0 (GraphPad, La Jolla, CA). A Wilcoxon matched test was used to analyze differences between 2 paired groups of data. Either a Friedman or a Kruskal-Wallis test was applied to analyze differences between more than 2 different groups. Correlation analysis was performed according to Spearman. A *P* value lower than .05 was considered statistically significant (∗*P* ≤ .05, ∗∗*P* ≤ .01, ∗∗∗*P* ≤ .001, ∗∗∗∗*P* ≤ .0001). All authors had access to the study data and had reviewed and approved the final manuscript.
